# The impact of the duration of the palliative care period on cancer patients with regard to the use of hospital services and the place of death: a retrospective cohort study

**DOI:** 10.1186/s12904-020-00547-8

**Published:** 2020-03-24

**Authors:** Outi M. Hirvonen, Riikka-Leena Leskelä, Lotta Grönholm, Olli Haltia, Samuli Voltti, Kristiina Tyynelä-Korhonen, Eeva K. Rahko, Juho T. Lehto, Tiina Saarto

**Affiliations:** 1 Department of Oncology and Radiotherapy, Turku University Hospital and Department of Clinical Oncology, University of Turku, PO Box 52, FI-20521 Turku, Finland; 2 Nordic Healthcare group, Helsinki, Finland; 3 Department of Palliative Care, Comprehensive Cancer Center, Helsinki University Hospital, and Faculty of Medicine, Helsinki University, Helsinki, Finland; 4 Tuusula Health Care Centre, Tuusula, Finland; 5 Center of Oncology, Kuopio University Hospital, Kuopio, Finland; 6 Department of Clinical Oncology, Oulu University Hospital, Oulu, Finland; 7 Department of Oncology, Palliative Care Unit, Tampere University Hospital and Faculty of Medicine and Health Technology, University of Tampere, Tampere, Finland

**Keywords:** Palliative care, Cancer, End-of-life care, Hospitalization, Emergency department, Place of death, Advance care planning, Finland

## Abstract

**Background:**

In order to avoid unnecessary use of hospital services at the end-of-life, palliative care should be initiated early enough in order to have sufficient time to initiate and carry out good quality advance care planning (ACP). This single center study assesses the impact of the PC decision and its timing on the use of hospital services at EOL and the place of death.

**Methods:**

A randomly chosen cohort of 992 cancer patients treated in a tertiary hospital between Jan 2013 –Dec 2014, who were deceased by the end of 2014, were selected from the total number of 2737 identified from the hospital database. The PC decision (the decision to terminate life-prolonging anticancer treatments and focus on symptom centered palliative care) and use of PC unit services were studied in relation to emergency department (ED) visits, hospital inpatient days and place of death.

**Results:**

A PC decision was defined for 82% of the patients and 37% visited a PC unit. The earlier the PC decision was made, the more often patients had an appointment at the PC unit (> 180 days prior to death 72% and < 14 days 10%). The number of ED visits and inpatient days were highest for patients with no PC decision and lowest for patients with both a PC decision and an PC unit appointment (60 days before death ED visits 1.3 vs 0.8 and inpatient days 9.9 vs 2.9 respectively, *p* < 0.01). Patients with no PC decision died more often in secondary/tertiary hospitals (28% vs. 19% with a PC decision, and 6% with a decision and an appointment to a PC unit).

**Conclusions:**

The PC decision to initiate a palliative goal for the treatment had a distinct impact on the use of hospital services at the EOL. Contact with a PC unit further increased the likelihood of EOL care at primary care.

## Background

Cancer patients are often admitted to hospital care during the last months of their life [1]. In some cases, this is unavoidable, but an increased number of emergency department (ED) visits, inpatient hospital admissions, or dying in an acute-care setting is also a characteristic of insufficient palliative care (PC) for patients with advanced cancer approaching their end-of-life (EOL) [2, 3]. In contrast, patients receiving in-home PC are less likely to visit the ED or to be admitted to a hospital than those receiving standard care [4, 5]. Furthermore, community-based palliative home-care services are not only associated with reduced ED visits, but also with fewer and shorter hospitalizations, lower risk of intensive care unit (ICU) admission, as well as an increased likelihood of a home death [6–9].

It has been demonstrated that early integrated PC leads to less aggressive EOL care, including reduced chemotherapy and longer hospice care [10]. The longer the hospice care period the better the quality of life [11]. Early integrated palliative care also reduces the rates of hospitalization and ED visits [10, 12]. Thus, palliative decision making is preponed.

Internationally, the terminology of the PC period and its timing and content is somewhat confusing. In the Lancet Oncology Commission paper [13] the terms are defined based on the treatment intention: curative, life prolonging, or palliative. However, PC can be integrated at any stage of the disease trajectory, irrespective of the primary intention of the treatment [13]. When the primary treatment goal is set to palliative, and the disease modifying treatments end, the period of PC begins. In our study this moment is called the PC decision.

An earlier transition into PC can be one way of reducing the use of hospital services at the EOL [2, 3]. It gives more time to initiate and carry out advance care planning (ACP) including a connection to primary care or a hospice as well as organizing care in the home, if possible. ACP is important; although it can be conducted by general practitioners, PC specialists are highly skilled in this area. Thus, an appointment at a PC unit may offer one approach for improving the quality and completion of the ACP documentation [14]. Consequently, the service needs of the patients at the EOL should be addressed outside both the ED and the secondary or tertiary care hospital. The site of death may then indicate the quality of end-of-life care, as the majority of patients with the serious illness want to die at home rather than in an institutional setting [11].

Although there are studies showing that an earlier introduction (of one to more than three months prior to death) of PC is associated with improved EOL care, in terms of fewer hospitalizations and increased likelihood of dying at home or in a hospice [15–18], there is no data, to our knowledge, regarding the impact of the transition from life-prolonging anticancer treatments to PC and its timing on the hospitalizations and site of death in cancer patients.

In our earlier study [19], we reported that PC decisions done within the last month prior to death were associated with anti-cancer treatments continuing until close to death, and the access to a PC unit becoming more unlikely. Therefore, a well-timed decision to initiate a palliative period might also be related to more appropriate treatment and resource usage at the EOL.

The aim of this study was to evaluate whether the PC decision and a referral to a PC unit have an impact on the use of hospital services at the EOL and on the place of death.

## Methods

### Cohort selection

This cohort consists of a sample of patients with a cancer diagnosis (ICD-10 C00-C96) who had received treatment in the Department of Oncology at Helsinki University Central Hospital (HUCH) between January 1 –December 31, 2013 and were deceased by December 31, 2014. The total number of patients fulfilling the criteria was 2 737 and of these, 992 were randomly selected for the study cohort from the hospital register. Randomization was done by sorting the patients in the order of their pseudonymized identifier, creating a random order. Finally 949 patients were included; 43 patients with non-malignant primary cause of death or pediatric patients were excluded. This retrospective study was done with the permission of the authorities of HUCH. According to Finnish legislation, no ethics committee approval was needed.

Finnish cancer patients are treated mainly at public university and central hospitals. HUCH is the largest university hospital in Finland being responsible for the cancer care of a population of approximately 1.6 million in Southern Finland. HUCH is governed by the representatives of all the municipalities in the region and HUCH provides all secondary and tertiary care for these municipalities. During the time of this study, the HUCH Department of Oncology provided radiation therapy treatments for all cancer patients and systemic cancer treatments for most cancer patients (not for pediatric (<18 years), hematological, gynecological or lung cancer patients). There is a PC outpatient unit in the Department of Oncology, but municipalities, who in Finland organize primary care, are responsible for EOL care. However, early integrated PC was not systematically provided at HUCH at the time of the study.

### Data sources and collection

The data and data sources used in the study are the same as in [19], but some new variables were considered in addition to those in [19]: do not resuscitate (DNR) decisions, visits to the ED in secondary/tertiary hospitals, inpatient episodes in secondary/tertiary hospitals, appointments to PC units, timing of the PC decision, and the date and place of death. Most data used in this study was available in a structured format and exported directly from the electronic medical records. Information on the PC decision, DNR decision and place of death were manually extracted by two of the authors (LH and OH). Due to the nature of the data, there were no missing values as it is mandatory to record all the parameters used. The only missing or imprecise information was in the death certificates concerning the place of death (3%).

The cancer diagnoses were grouped in the same way as in [19] into 13 groups. When the patient had more than one malignancy, the cancer diagnosis was recorded to match the primary cause of death.

### Division of categories and service usage

The service usage of all patients was studied 14, 30, and 60 days before the time of death. Service usage is enumerated by two measures, the number of visits to an emergency department (‘ED visits’) and the number of nights spent in the hospital (‘inpatient days’). The places of death were categorized in five categories: home, primary care wards, secondary or tertiary care wards, hospices, and nursing homes. In the 24 municipalities of Southern Finland, at the time of the study, there was one hospice, one PC ward in primary care and seven home care teams specialized in PC.

The PC decision and the PC period are defined as in [19]. For the purposes of this study patients were divided into three separate categories: ‘no PC decision’, ‘PC decision’, and ‘PC decision and appointment to a PC unit’. This definition is operated in a dynamic fashion in the analyses to ensure a correct chronology of events. For example, if patients have a PC decision made 40 days prior to death and have visited the PC unit 20 days before death, they will be categorized as ‘PC decision’ for the analysis of events 30 days prior to death, and as ‘PC decision and appointment to a PC unit’ for the analysis of events 14 days prior to death. However, when considering service usage 60 days prior to death, they will be categorized as ‘no PC decision’, as at that time neither the PC decision nor the appointment have taken place. Thus, in each analysis, the patients were categorized depending on the timing of the PC decision and PC unit appointment with respect to the time period studied.

### Statistical analysis

Means, standard deviations, and distributions were used for patient characteristics. The frequency of DNR decisions and the distribution of the places of death were analyzed by cross-tabulation. Pairwise Pearson’s chi squared tests were conducted to statistically test for the differences between the three categories with respect to DNR and place of death. The difference between the three categories with respect to ED and inpatient service usage was tested with pairwise t-tests (pooled standard deviation and p-values adjusted with the Holm method). The association of the PC decision and PC unit appointments with hospital service usage was also tested with linear regression models including control variables (age, time from diagnosis to death, and cancer diagnosis). Logarithmic transformations for the dependent and independent variables were conducted to normalize the residuals in the regression models. All analyses were performed using R-studio version 1.1.447 and its packages.

## Results

Characteristics of the patients are presented in Table 1. For most patients (82%) a PC decision was made, and 37% of the patients had an appointment at the PC unit. The frequency of DNR decisions in the ‘No PC decision at all’ category is statistically significantly smaller than in the ‘PC decision at some point’ category (*p*< 0.01), as is the frequency of DNR decisions in the ‘PC decision and appointment to a PC unit at some point’ category when compared to the ‘PC decision at some point’ category (*p*<0.05). However, the differences in the frequency of DNR decisions is not statistically significant between the ‘No PC decision at all’ and the ‘PC decision and appointment to a PC unit at some point’ categories. The pairwise comparisons of the distributions of the places of death between the three patient categories are all statistically significant (*p*<0.01).

**Table 1:** Characteristics of the patients

	Category			
Measure	No PC decision at all	PC decision at some point	PC decision and appointment to a PC unit at some point	Total
Number of patients, N (%)	176 (19%)	424 (45%)	349 (37%)	949 (100%)
Gender, N (%)				
Male	86 (49%)	225 (53%)	188 (54%)	499 (53%)
Female	90 (51%)	199 (47%)	161 (46%)	450 (47%)
Age (years) at death, mean (Stdev)	64 (11.9)	67 (11.8)	68 (12.5)	67 (12.1)
Cancer diagnoses, N (%)				
Upper gastrointestinal	29 (16%)	83 (20%)	106 (30%)	218 (23%)
Colorectal cancers	17 (10%)	52 (12%)	54 (15%)	123 (13%)
Lung *	27 (15%)	75 (18%)	16 (5%)	118 (12%)
Breast cancer	24 (14%)	53 (13%)	29 (8%)	106 (11%)
Prostate cancers	13 (7%)	19 (4%)	35 (10%)	67 (7%)
Cancers of urinary tract	6 (3%)	29 (7%)	26 (7%)	61 (6%)
Primary CNS malignancies	16 (9%)	30 (7%)	14 (4%)	60 (6%)
Lymphomas	6 (3%)	20 (5%)	11 (3%)	37 (4%)
Invasive skin cancers	6 (3%)	17 (4%)	9 (3%)	32 (3%)
Sarcomas	5 (3%)	10 (2%)	15 (4%)	30 (3%)
Gynecological cancers *	9 (5%)	8 (2%)	11 (3%)	28 (3%)
Head & Neck (H&N)	8 (5%)	12 (3%)	8 (2%)	28 (3%)
Others	10 (6%)	16 (4%)	15 (4%)	41 (4%)
Time (months) from diagnosis to death, mean (Stdev)	33 (44)	37 (43)	41 (46)	37 (44)
DNR decision made, N (% of category)	77 (44%)^a,c^	240 (57%)^a,b^	171 (49%)^b,c^	488 (51%)
Place of death, N (%)				
Home	26 (15%)	37 (9%)	73 (21%)	136 (14%)
Hospice	10 (6%)	53 (13%)	31 (9%)	94 (10%)
Nursing home	3 (2%)	9 (2%)	7 (2%)	19 (2%)
Primary care ward	81 (46%)^d^	236 (56%)^d^	205 (59%)^d^	522 (55%)
Secondary / tertiary healthcare	50 (28%)^d^	80 (19%)^d^	22 (6%)^d^	152 (16%)
Unknown	6 (3%)	9 (2%)	11 (3%)	26 (3%)

* only patients receiving radiotherapy are included

^a^ The difference between these groups is statistically significant (*p* < 0.01)

^b^ The difference between these groups is statistically significant (*p* < 0.05)

^c^ The difference between these groups is not statistically significant (*p* > 0.05)

^d^ The pairwise differences between these groups are statistically significant (*p* < 0.01)

### Place of death

The association of the PC decision and the PC unit appointment with the place of death is also presented in Table 1. The significant difference between the categories is that patients with no PC decision died more often in secondary/tertiary care wards (28% vs. 19% and 6%, respectively) whereas patients with a PC decision or both a PC decision and a PC unit appointment were more likely to die in primary care wards (46% vs. 56% and 59%, respectively).

### Resource use

Table 2 describes the timing of the PC decision with respect to the time of death, and the proportion of patients visiting the PC unit. The earlier the PC decision was made, the more often patients also visited the PC unit.

**Table 2:** Timing of the palliative care decision and the proportion of patients visiting a PC unit (%)

Time between the PC decision and death	All patients with PC decision N (% of all patients)	Patients with PC outpatient unit appointment N (% patients with PC decision within the timeframe)
< 14 days	171 (22%)	17 (10%)
14–30 days	141 (18%)	50 (35%)
31–90 days	206 (27%)	105 (51%)
91–180 days	113 (15%)	75 (66%)
> 180 days	142 (18%)	102 (72%)

**Fig. 1:**
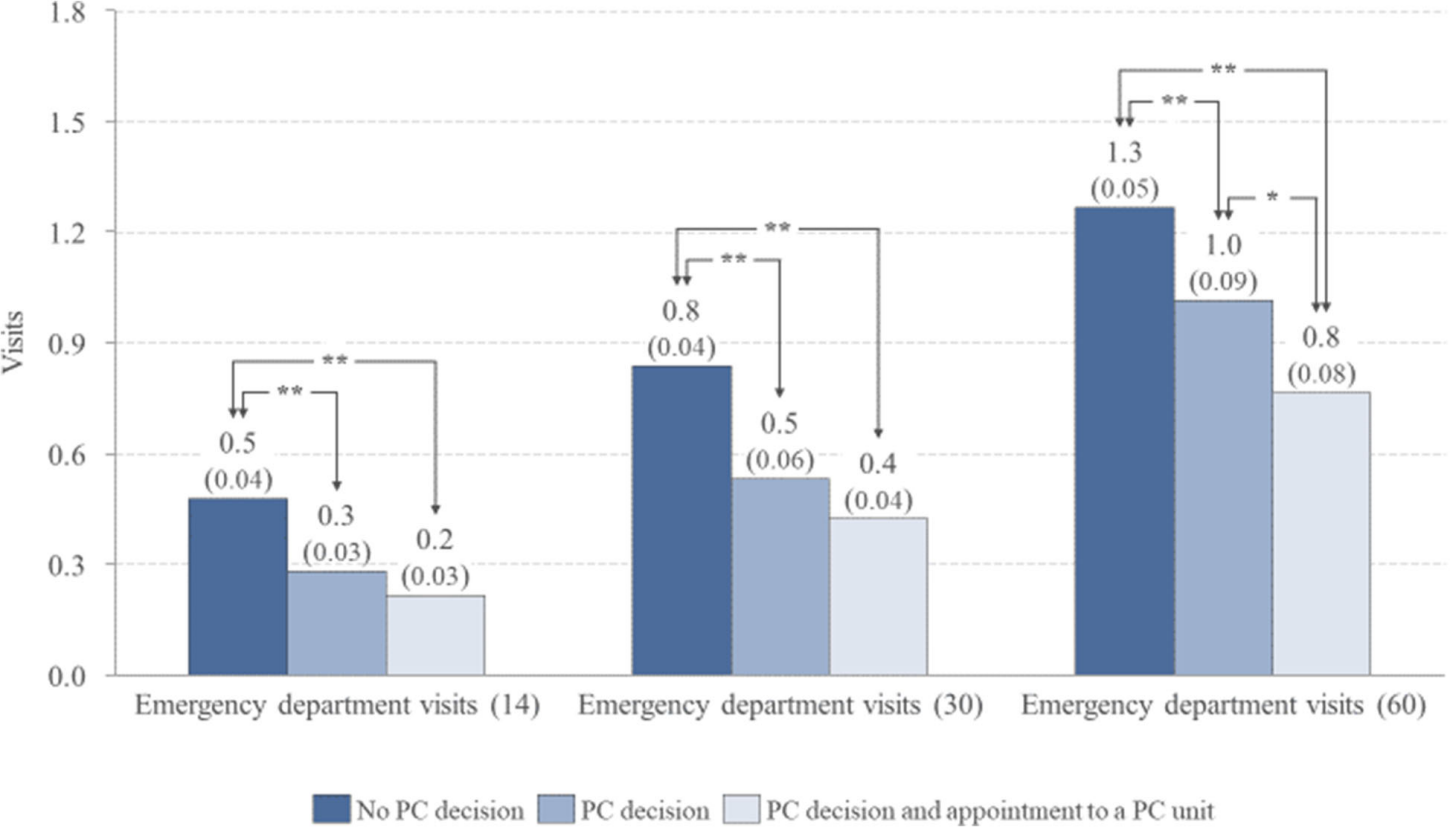
Mean number of emergency department visits 14, 30, and 60 days before the death of patients with no PC decision, with a PC decision and with both a PC decision and visit to a PC unit before the time frame under consideration. Standard errors of the mean in parentheses. * The pairwise comparison of the mean number of inpatient days is statistically significant (*p* < 0.05) ** The pairwise comparison of the mean number of inpatient days is statistically significant (p < 0.01)

**Fig. 2:**
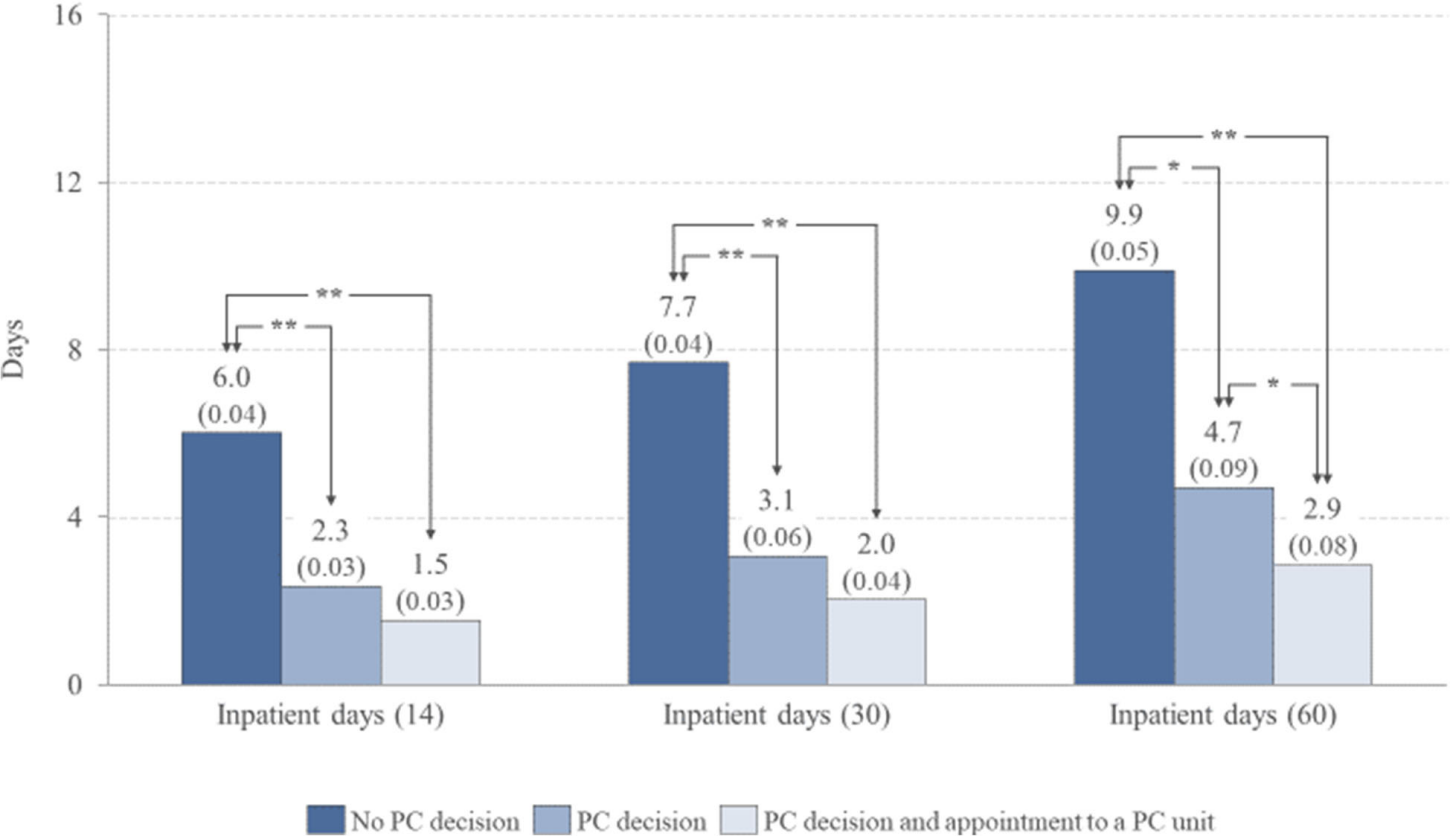
Mean number of inpatient days 14, 30, and 60 days before the death of patients with no PC decision, with a PC decision and with both a PC decision and a visit to a PC unit before the time frame under consideration. Standard errors of the mean in parentheses. * The pairwise comparison of the mean number of inpatient days is statistically significant (*p* < 0.05) ** The pairwise comparison of the mean number of inpatient days is statistically significant (*p* < 0.01)

Figures 1 and 2 depict the average resource usage of the three categories of patients in the hospital ED and inpatient wards 14, 30 and 60 days prior to death. The average number of ED visits (Fig. 1) and the average number of inpatient days (Fig. 2) were highest for patients with no PC decision and lowest for patients with both a PC decision and a visit to a PC unit.

The results of the regression models are presented in Table 3. The models confirm the negative association between the PC decision and service usage prior to death, as well as between PC unit appointments and service usage prior to death, even after considering potential control variables, such as age and cancer site. On average, patients with a PC decision and an appointment to a PC unit had 15-24% less ED visits and 56-64% fewer inpatient days than the patients without a PC decision.

**Table 3:** Results of the regression model expressed as the proportional change (%) in the patients’ services usage 14, 30 and 60 days prior to death resulting from an increase in the covariates by 1 %. In the case of binary variables, the coefficient depicts the proportional changed in the service in each diagnosis group compared to breast cancer patients

	Dependent variable					
	Emergency department visits			Inpatient days		
**Number of days before death**	**14**	**30**	**60**	**14**	**30**	**60**
Age	− 0.2^a^	− 0.1	− 0.2^b^	−0.3	− 0.1	−0.4^b^
Days from diagnosis to death	0.0	0.0	0.0	0.0	−0.1	− 0.1
Binary variable: Diagnosis groups (reference category: Breast cancer)						
Cancers of urinary tract	−0.4	1.0	20.2^b^	9.2	25.8	42.7
Colorectal cancers	4.0	7.9	9.1	−5.1	16.9	10.4
Gynecological cancers	3.2	−1.6	−8.7	1.3	11.7	25.9
Head & Neck (H&N)	−10.2	− 16.7	− 18.5	−30.6	−29.8	− 8.1
Lung	8.7	10.8	7.9	9.0	12.8	13.2
Lymphomas	−1.9	2.0	5.7	50.8^b^	60.3^b^	64.2^b^
Melanoma and other skin cancers	−0.6	−4.9	−7.9	−24.1	− 26.2	−23.8
Others	−0.6	5.8	5.3	33.4	81.9^a^	89.6^a^
Primary CNS malignancies	−11.7^b^	−17.8^a^	−22.1^a^	−44.1^a^	−38.1^a^	−39.4^a^
Prostate cancers	6.6	10.3	13.6	7.6	12.2	15.4
Sarcomas	−11.7	−19.2^b^	−19.7	− 32.7	−24.7	−25.6
Upper gastrointestinal	6.8	8.7	12.2	5.1	22.1	20.6
Binary variable: PC categories (reference category: ‘no PC decision’)						
PC-decision	−12^a^	−18.6^a^	−13.4^a^	−47.1^a^	−57.6^a^	−51.1^a^
PC-decision and admission to PC unit	−15.3^a^	−21.9^a^	−24.2^a^	−56^a^	−63.4^a^	−64.4^a^
**Test statistics**						
Adjusted R^2^	0.06	0.08	0.06	0.13	0.18	0.16
F-stat	4.45	6.13	4.98	10.16	14.16	12.02
Residual standard error	0.35	0.44	0.53	1.10	1.08	1.14

^a^Significant at 0.01, ^b^Significant at 0.05

## Discussion

In this assessment of the treatment of cancer patients at a Finnish university hospital, a PC decision to initiate a palliative intention for the treatment decreased the number of ED visits and inpatient days in a secondary/tertiary care hospital. Patients without a PC decision also died more often in a secondary or tertiary care hospital compared to the patients with a PC decision. The usage of health care services was further decreased if the patient had an appointment to a PC outpatient unit in addition to a PC decision.

These results give an indication that a PC decision and an appointment to a PC outpatient unit may improve the quality of EOL care. However, it must be kept in mind that this conclusion applies on an aggregate level; on the individual level ED visits and hospitalizations may still be very well justified.

Internationally, the terminology of the PC period and its timing and content are not uniform. We chose to divide patients according to the timing of PC decisions and service usage by using time periods before death on clinical grounds and thus complying with most of the previous studies; however, the lack of strict recommendations on this timing makes comparison between previous studies and the present study somewhat difficult. Regarding the timing of the PC initiation, in two previous studies [15, 17] early PC referral was defined as a referral to PC ≥30 days, whereas in the study by Alsirafy and co-workers [16] the timing of PC referrals were categorized as early (> 90 days before death), intermediate (30-90 days before death) and late (<30 days before death). In the study by Nieder and co-workers [18], three months before death was chosen as a time point to distinguish between an early and late PC. However, the timing of the PC period should be considered together with the content of the PC period, that is, whether patients received only treatments managing symptoms, or also disease-modifying treatments. These studies have not made a distinction between the patients receiving early integrated PC (during the active oncologic treatments), or late PC (after discontinuation of active oncologic treatments). No systematic early integrated PC was offered at HUCH during the time period of this study, although early integration has since been recommended by the clinical practice guidelines of the American Society of Clinical Oncology (ASCO), as well as the Lancet Commission of integration of oncology and PC [13, 20]. Therefore, our cohort only represents PC introduced after the termination of anti-cancer treatments; although the current evidence and recommendations highlight the importance of providing PC early and integrating it with disease modifying therapies. It is due to this limitation and the fact that most of the PC decisions were made quite close to death (for 67 % of patients who had a PC decision, it was made within the last 3 months of life) that we chose to study the effect of the PC decision and the PC unit appointment with binary (yes/no) variables. This approach loses some information compared to an approach using the exact time lags between the PC decision and PC unit appointment and death in the statistical modeling. However, using this approach meant that the results were more robust and statistical significance could be detected.

We have earlier demonstrated with this same cohort of patients [19] that a PC decision initiating the palliative goal for care was frequently made, but occurred late [11]: the median time from the PC decision to death was 46 days. Patients with no or a very late PC decision (<30 days) received more aggressive cancer treatments at the EOL and made fewer visits to the PC unit. Only 37% of these patients visited the PC unit. In this study, we further show that patients with no or very late PC decision not only receive more aggressive treatments at the EOL but also use more hospital services and have a higher risk of dying in an acute-care setting. These are often considered to be indicators of a poor quality of end of life care [21].

Despite the introduction of PC only after discontinuation of active oncological treatments in our cohort, we did observe the benefit of PC decisions in reducing the ED visits and inpatient days in the hospital, especially during the last months of life. A referral to the PC outpatient unit further decreased the ED visits and inpatient days. The reduction in the average number of inpatient days per patient was significant (5-7 days per patient). The reduction in the average number of ED visits per patient was 0.3 – 0.5 visits. Even a small reduction in the number of ED visits, however, is significant in economic terms: for this study population it would mean approximately 200 fewer ED visits during the last month of life. In addition, we believe that every unnecessary visit to an ED during the last weeks of life are burdensome for the patient. However, the coefficients of determination (adjusted R2) of the regression models are relatively low – especially in the model for ED visits – indicating that there is considerable variation in the data that this model does not capture. A systematic review and meta-analysis has indicated that PC services decrease the likelihood of ED visits [3]. An earlier study of cancer patients in Finland also showed that a visit to the PC outpatient clinic facilitated the connection with primary care services and tended to decrease ED visits and resource usage of the tertiary care hospital [14]. Both these earlier studies are in line with our results. We suggest that although a switch to a palliative goal for care may modify the EOL care arrangements of a patient, a sufficiently early contact with the PC services might have an even higher impact [10].

Early PC (initiated > 1 to > 3 months prior to death depending on the study) has been shown to be associated with fewer hospitalizations, earlier DNR designation, and an increased likelihood of dying at home or in a hospice instead of a hospital or an ICU [15–18]. In line with the previous studies, in our study, patients without a PC decision and especially those without any contact to the PC unit were more likely to die in a secondary or tertiary hospital ward. However, in the present study, the majority of the patients died in a primary care ward as there were only a few hospices or wards specializing in EOL care available during that time. Likewise, dying at home was rare due to the lack of specialized palliative home care teams.

Patients dying of cancer use the resources of hospitals - often for a good reason – but any reduction in the utilization of hospital wards is also beneficial from an economic perspective [22]. Indeed, it has been shown that palliative home care support or a proactive PC program reduces hospital use and the total costs of care at the EOL [9, 23]. We did not carry out a cost benefit analysis in this study, but this important aspect warrants investigation in future studies.

There are some limitations to our study. One limitation is that the study was conducted on a random sample of the entire cohort. However, the sample of about 1000 patients was considered large enough to detect statistically significant differences between the patient groups, as this ensured that there would be over 100 patients in all the categories that were critical for the study (the number of patients with no PC decision, and patients with PC decision and an appointment to the PC unit). A random sample was chosen instead of the entire cohort because the manual collection of data from unstructured texts in medical records and death certificates proved so time-consuming that we would have needed more than two experts for the data collection. We considered the division of data collection among several experts to be a risk for the homogeneity of the data, and therefore chose to proceed with a sample.

In addition, the results do not contain the number of ED visits or inpatient days in primary care services since this information is not in the hospital databases. Furthermore, we could not exclude the possibility of some sudden deaths or deaths due to complications in the secondary or tertiary hospital; however, according to previous studies this would explain only a small proportion of deaths [24]. The lack of data on the quality of life is also a limitation. Finally, the analysis exploits the “benefit of hindsight” where the date of death is known. Thus, making recommendations on this basis for the future care of living patients is not possible without existing accurate prognostication as a part of clinical practice. The strength of the study is a relatively large sample size. The study cohort represent a population-based real-life situation comparable to epidemiological incidence of oncological diseases found within the population.

The retrospective chart review is one of the most reliable forms of research into PC and associated outcomes, due to the very high recruitment rate: once the approach is set and agreed, the desired number of cases can be gathered with very few exceptions.

## Conclusions

Our study revealed that although for most cancer patients a PC decision (i.e. the decision to alter the treatment goal to palliative) was made for less than half of the patients, the decisions made were in collaboration with a PC team. The lack of a PC decision or postponing it to the last weeks of life reflected a significantly increased risk of visits to an ED, more inpatient days in a secondary or tertiary care hospital, and further, dying in a secondary or tertiary care ward. Early integrated PC should be offered more systematically to ensure a timely ACP and access to palliative and EOL care.

## Data Availability

All data and material related to the manuscript have been archived and maintained by the authors at the University hospital of Helsinki, according to organizational and ethical regulations. The raw data of this article is archived by the corresponding author and will not be published so as to preserve patients´ privacy. Upon request the authors will share the data in suitable way.

## Abbreviations

PC: Palliative care
EOL: End-of-life
ED: Emergency Department
ACP: Advance care plan
DNR: Do-not-resuscitate
ICU: Intensive care unit
HUCH: Helsinki University Central Hospital
